# NHERF1/EBP50 is an organizer of polarity structures and a diagnostic marker in ependymoma

**DOI:** 10.1186/s40478-015-0197-z

**Published:** 2015-03-08

**Authors:** Maria-Magdalena Georgescu, Paul Yell, Bret C Mobley, Ping Shang, Theodora Georgescu, Shih-Hsiu J Wang, Peter Canoll, Kimmo J Hatanpaa, Charles L White III, Jack M Raisanen

**Affiliations:** Department of Pathology, The University of Texas Southwestern Medical Center, Dallas, TX 75390 USA; Department of Neuro-Oncology, The University of Texas MD Anderson Cancer Center, Houston, TX 77030 USA; Department of Pathology, Vanderbilt University Medical Center, Nashville, TN 37232 USA; Department of Pathology, Columbia University, New York, NY 10032 USA

**Keywords:** NHERF1/EBP50, Ependymoma, Hydrocephalus, Polarity, Moesin, PTEN

## Abstract

NHERF1/EBP50, an adaptor protein required for epithelial morphogenesis, has been implicated in the progression of various human malignancies. *NHERF1*-deficient mice have intestinal brush border structural defects and we report here that they also have disorganized ependymal cilia with development of non-obstructive hydrocephalus. Examination of mouse and human brain tissues revealed highest NHERF1 expression at the apical plasma membrane of ependymal cells. In ependymal tumors, NHERF1 expression was retained in polarized membrane structures, such as microlumens, rosettes and canals, where it co-localized with some of its ligands, such as moesin and PTEN. Analysis of a comprehensive panel of 113 tumors showed robust NHERF1 labeling of microlumens in 100% of ependymomas, subependymomas, and pediatric anaplastic ependymomas, and in 67% of adult anaplastic ependymomas. NHERF1 staining was present in 35% of ependymoma cases that lacked reactivity for EMA, the routine immunohistochemical marker used for ependymoma diagnosis. NHERF1 labeling of microlumens was either absent or rarely seen in other types of brain tumors analyzed, denoting NHERF1 as a reliable diagnostic marker of ependymal tumors. Anaplastic foci and a subset of adult anaplastic ependymomas showed complete absence of NHERF1-labeled polarity structures, consistent with a loss of differentiation in these aggressive tumors. These data highlight a role for NHERF1 in ependymal morphogenesis with direct application to the diagnosis of ependymal tumors.

**Electronic supplementary material:**

The online version of this article (doi:10.1186/s40478-015-0197-z) contains supplementary material, which is available to authorized users.

## Introduction

In the development of the central nervous system (CNS), ependymal cells arise from the asymmetric division of radial glia, the polarized precursor cells spanning the central canal to the pia mater [1,2]. Ependymal cells are terminally differentiated glial cells that line the ventricular system and retain the polarity of their precursors. Arranged as a single layer of cuboidal cells with adherens junctions, basally located nuclei and specialized apical plasma membrane (PM) containing cilia and microvilli, they resemble epithelial cells forming glandular lumens except for the lack of a well-defined basement membrane. Ependymal cells form the cerebrospinal fluid (CSF)-brain barrier and, through the unidirectional beat of their cilia, they direct the flow of the CSF in the ventricular system. Pathologically, structural alterations of the ependymal cilia may lead to hydrocephalus development [3], whereas the uncontrolled proliferation of ependymal cells or their precursors results in the growth of generally non-infiltrative glial ependymal tumors. Depending on their mitotic rate, ependymal tumors fall into three categories, in increasing order of aggressiveness: subependymomas (WHO grade I), ependymomas (WHO grade II) and anaplastic ependymomas (WHO grade III). Whereas subependymomas occur in adults and have a benign course, higher grade tumors are more likely to arise in childhood where overall 5-year survival rates of 60% are seen [4]. These rates have not improved significantly in the past 40 years due to lack of effective chemotherapy and a poor understanding of tumor pathogenesis [5].

NHERF1/EBP50 (Na^+^/H^+^ exchanger 3 regulating factor 1; ezrin-radixin-moesin (ERM) binding phosphoprotein 50) is an adaptor protein localized mainly at the apical PM in human epithelia [6]. NHERF1 interacts with the ERM-NF2 (neurofibromatosis 2) cytoskeletal proteins via its carboxyl (C)-terminal ERM-binding region and with many ligands, including PTEN tumor suppressor and platelet-derived growth factor receptor (PDGFR), via its amino (N)-terminal tandem PDZ (PSD95-Dlg1-ZO1) domains [7,8]. NHERF1 behaves as a tumor and epithelial-to-mesenchymal transition suppressor in cultured cells, through its effects on PTEN and β-catenin [9-11], and is required for gland morphogenesis with lumen formation in three-dimensional polarized epithelium [12]. Importantly, NHERF1 overall loss or PM displacement has been reported in aggressive tumors, including carcinomas and glioblastoma [9,13,14]. *NHERF1* knockout mice have ultrastructural defects of the intestinal apical brush border membrane and of the cochlear outer hair cell cilia bundles [15,16]. Prompted by the observation that these mice also develop non-obstructive hydrocephalus, we mapped the highest NHERF1 expression in the CNS at the specialized apical PM of ependymal cells. The immunohistochemical of ependymal tumors showed a unique expression of NHERF1 and some NHERF1-associated molecules, such as moesin, in microlumens that represent precursor polarized membrane structures retained by neoplastic ependymal cells. Besides this robust and specific NHERF1 expression that we propose as a diagnostic marker for these tumors, a gradual loss of NHERF1 was observed in anaplastic ependymomas, compatible with a previously demonstrated tumor suppressor role for NHERF1.

## Materials and methods

### Animals

The *NHERF1*-deficient mice were inbred for 10 generations in C57BL/6J background and genotyped as described [16]. Newborn mice were observed regularly for skull deformity or signs of neurological impairment. When skull deformity was present, mice were sacrificed and the skulls were dissected and grossly examined by serial sectioning following decalcification. To assess the incidence of subclinical hydrocephalus, whole litters of 5 week-old progeny from *NHERF1* heterozygous parents were examined as above. All experiments were performed under approved MD Anderson Cancer Center IACUC protocols.

### Mouse tissue histology and immunostaining

Brains were fixed overnight in 10% formalin, embedded in paraffin and 4μm sections were processed for hematoxilin and eosin (H&E) staining as described [16]. The immunofluorescence analysis was performed as described [11] with NHERF1 1:300 (Abcam, Cambridge, MA), β-catenin 1:500 and acetylated α-tubulin 1:1000 (Sigma-Aldrich, St Louis, MO) primary antibodies. Image stacks were acquired with a Zeiss Axiovert 200M inverted microscope (Carl Zeiss MicroImaging, Thornwood, NY) and deconvolved with AxioVision Rel 4.5 SP1 software.

### Human specimens, histology and electron microscopy

Brain tumor resection or biopsy specimens were obtained from the Division of Neuropathology University of Texas Southwestern Medical Center, Dallas, TX, Division of Neuropathology, Columbia University, New York, NY and Department of Pathology, Vanderbilt University, Nashville, TN. The specimens were processed for H&E staining or immunohistochemistry (IHC) [17], with antibodies for NHERF1 1:3200 (Thermo/Fisher, Waltham, MA), moesin 1:100, PTEN 1:100 and PDGFRα 1:100 (Cell Signaling Technology, Danvers, MA), NF2 1:200 and YAP1 1:200 (Santa Cruz Biotechnology, Santa Cruz, CA), β-catenin 1:1600 (Invitrogen, Carlasbad, CA), EGFR 1:1000 and EMA 1:400 (Dako, Carpinteria, CA), and PHLPP2 1:100 (Bethyl Laboratories, Montgomery, TX).

### Statistical analysis and scan imaging

Images were acquired at 20x magnification, and where specified, at 40x magnification, with Aperio Scanscope CS2 whole slide image system (Leica Biosystems, San Diego, CA), analyzed by ImageScope software, version 12.1.0.5029, and quantified using the Nuclear algorithm, version 11.2. Three tumor areas were analyzed from each slide. When multiple tumor fragments were present, areas from 3 different fragments were chosen. The Nuclear algorithm was fine-tuned for object recognition, including intensity thresholds, edge trimming of objects and smoothing/declustering of nuclei/lumens, in order to obtain the primary output represented by the number of positive lumens and number of negative nuclei. Numerical data were examined for normality of distribution and expressed as mean ± SEM by using the GraphPad Prism program (GraphPad Software, La Jolla, CA). Two-tailed t-test with Welch’s correction for variances significantly different was used to analyze the differences between groups. Statistical significance was considered for P < 0.05. Confidence intervals for all tests were 95%.

## Results

### *NHERF1*-deficient mice develop hydrocephalus

**Figure 1:**
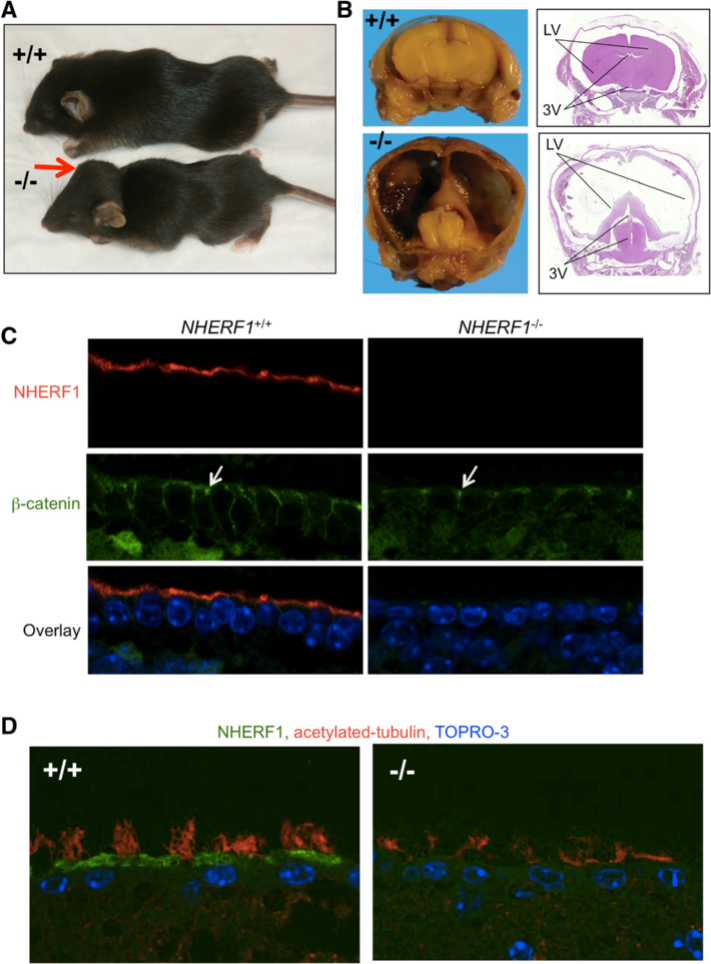
***NHERF1***-**deficient mice develop hydrocephalus. A**-**B**. Comparison between 5-week-old *NHERF1*(+/+) and (−/−) littermates showing smaller size and bossed skull (arrow) (A) and severely distended 3^rd^ (3V) and lateral ventricles (LV) with compression of the brain, resulting in a thin nutshell appearance of the cerebral hemispheres **(B)** in the *NHERF1*(−/−) littermate. **C**. Immunoflourescence analysis of 5-week-old *NHERF1*(+/+) mice and their *NHERF1*(−/−) littermates with subclinical mild hydrocephalus shows NHERF1 labeling of the apical PM in *NHERF1*(+/+) ependyma and β-catenin labeling of adherens junctions (arrows) in both genotypes. **D**. Acetylated tubulin immunofluorescence labeling of the ependymal cilia shows robust cilia tufts in *NHERF1*(+/+) mice and present but disorganized cilia in *NHERF1*(−/−) littermates with subclinical mild hydrocephalus.

We and others have characterized a series of morphological and phenotypical alterations in *NHERF1*-deficient mice that include phosphate reabsorption impairment, altered intestinal brush border membrane, lack of development of the lobuloalveolar lactating mammary gland and abnormalities of cochlear cilia bundles with hearing defects [15,16,18,19]. A proportion of *NHERF1*-deficient mice also showed dilatation of the lateral, 3rd and 4th ventricles of the brain that define non-obstructive hydrocephalus (Figure 1A-B). The degree of hydrocephalus varied from overt forms, in which skull deformity and severe developmental impairment were present (Figure 1A), to clinically inapparent forms, in which mild to moderate dilatation of the ventricles was observed after skull dissection and brain sectioning (Additional file 1: Figure S1A). Wheareas overt forms were sporadic, the dissection of several litters generated from *NHERF1* heterozygous parents showed a variable penetrance of the mild phenotype with rates up to 100% (Additional file 1: Figure S1B).

Examination of NHERF1 expression in brain sections showed that NHERF1 is most highly expressed at the apical PM of ependymal cells, followed by lower expression levels in choroid plexus cells (Additional file 1: Figure S2). Both cells types have been involved in the development of non-obstructive hydrocephalus, either through impaired cilia motion in the case of ependymal cells [3] or by hyperproduction of CSF, usually in choroid plexus hyperplasia [20]. Since we did not observe choroid plexus hyperplasia in *NHERF1*-deficient mice, we further characterized the ependymal cells. In overt hydrocephalus forms, the ependymal layer was flattened and disrupted (Additional file 1: Figure S3), most likely secondary to increased CSF pressure. In the mild forms, co-staining with NHERF1 and β-catenin antibodies showed only minor flattening of the ependymal layer and preservation of the lateral cell-cell adherens junctions (Figure 1C). In the latter forms, labeling of the ependymal cilia with acetylated tubulin antibody showed cilia disorganization in *NHERF1*-deficient animals as compared to the prominent tufts of cilia observed in wild-type animals (Figure 1D). These results suggested an involvement of NHERF1 in structuring the apical PM of ependymal cells by controlling cilia distribution.

### NHERF1 labels polarity membrane structures in ependymal tumors

**Figure 2:**
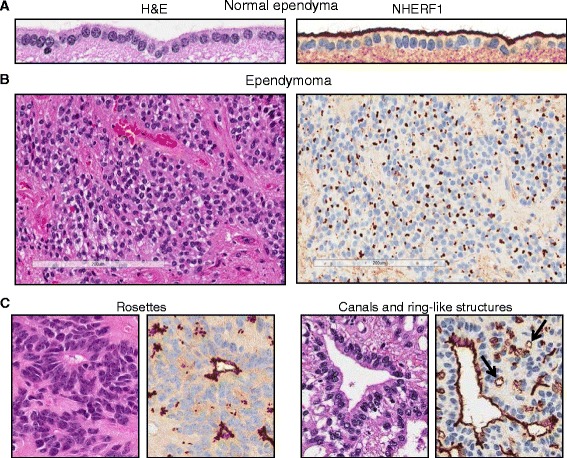
**NHERF1 labels polarity membrane structures in ependymoma. A**-**B**. IHC with NHERF1 antibody highlights the apical PM of human normal ependyma **(A)** and microlumens in ependymoma **(B)**. **C**. Serial sections from ependymoma cases stained with H & E and NHERF1 show apical PM labeling of rosettes, canals and ring-like structures (arrows) by NHERF1.

The association between the intestinal morphogenetic function of NHERF1 in *NHERF1*-deficient mice and an oncogenic function in human colorectal cancer [9,16], sugested the possibility of a parallel association between a structural role of NHERF1 in ependymal apical PM organization and the pathogenesis of ependymal tumors. To verify this hypothesis, we confirmed that the high ependymal apical PM expression found in mouse CNS is present in human CNS (Figure 2A). We have previously shown that the NHERF1 apical PM expression from colonic epithelial cells is lost early in the progression of colorectal cancer [9]. Strikingly, in ependymal tumors NHERF1 remained prominently retained in perinuclear dot-like structures (Figure 2B) that correspond ultrastructurally to microlumens, polarized structures characteristic for neoplastic cells of ependymal origin. Microlumens are delineated by a membrane containing the same specialized structures, microvilli and occasionally cilia, as the polarized apical PM of non-neoplastic ependymal cells. Ring-like structures, deemed to be specific for ependymoma [21], and likely representing larger microlumens, were also labeled by NHERF1 (Figure 2C, arrows).

Histologically, a minority of ependymomas harbor characteristic arrangements of neoplastic cells in rosettes delimiting a lumen (“true rosettes”) or, more rarely, in canals where they mimic closely the polarized lining of the ventricles. NHERF1 labeled the apical PM of true rosettes and canals (Figure 2C), indicating that in ependymoma NHERF1 specifically labels polarized structures which include a membrane-bordered lumen.

### NHERF1 organizes protein complexes with moesin and PTEN in ependymal polarity structures

**Figure 3:**
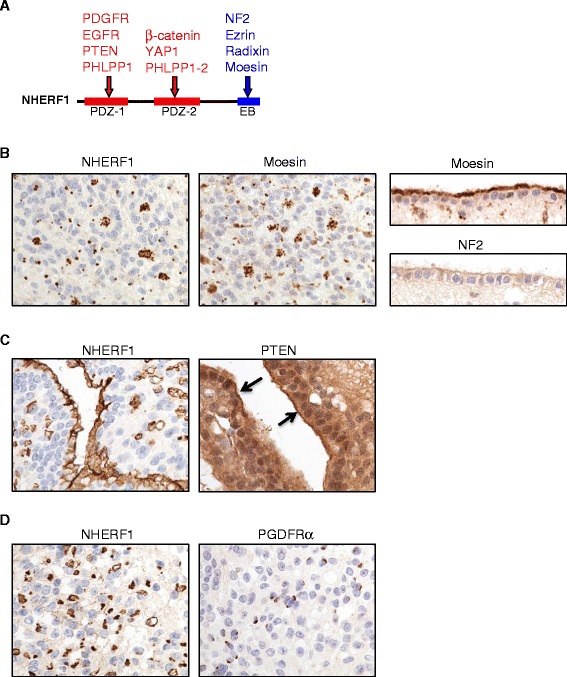
**NHERF1, moesin, and PTEN localize to ependymal polarized structures. A**. Schematic NHERF1 structure shows the N-terminal PDZ domains (1 and 2) and the C-terminal ERM-binding (EB) region with selected ligands. **B**. Serial sections from an ependymoma case showing localization of NHERF1 and moesin to microlumens and rosettes. Normal ependyma shows apical PM labeling with moesin, similar to NHERF1 but not with NF2 (right panels). **C**-**D**. Serial sections from an ependymoma case show the apical PM of canals labeled by NHERF1 and PTEN (arrows) antibodies (C) and NHERF1 microlumens distinct from the PDGFRα punctate or linear perinuclear staining **(D)**.

NHERF1 establishes protein complexes at the apical PM of epithelia that are essential for apico-basal polarity [12,16]. To investigate the composition of NHERF1 protein complexes in ependymoma, we screened the intracellular localization of the NHERF1 ligands moesin, NF2, PTEN, PDGFRα, EGFR, YAP1, β-catenin and PHLPP2 that have been functionally involved in primary brain tumors [11,22-27] (Figure 3A). The apical PM of normal ependyma and the various ependymoma polarity structures, including microlumens, rosettes, and canals, consistently showed PM localization of moesin, similar to NHERF1 (Figure 3B). *NF2* somatic mutations are the most frequent individual gene mutations in spinal cord ependymomas, where they reach 43% [28]. NF2 IHC in normal ependymal lining and ependymomas showed only faint or no labeling of apical PM (Figure 3B), respectively, suggesting that NF2, unlike moesin, is not a major ligand of NHERF1 in these polarized structures.

Among the NHERF1 PDZ-domain ligands, PTEN was detected at the apical PM of ependymal polarity structures similar to NHERF1 (Figure 3C). The major fraction of PTEN was cytoplasmic, as previously described [12,29]. Other NHERF1 ligands, such as PDGFRα, localized in sparse perinuclear dot-like or cap-like structures that appeared to be distinct from the NHERF1-labeled microlumens (Figure 3C). This PDGFRα staining, most likely associated with the Golgi apparatus, was focal in ependymomas and was also present in the NHERF1-negative anaplastic ependymoma and anaplastic astrocytoma cases screened. EGFR was not detected in ependymoma. The NHERF1 PDZ2 domain ligands β-catenin and YAP1 had a strong and diffuse cytoplasmic localization (Additional file 1: Figure S4). YAP1 also displayed nuclear staining, most prominent in anaplastic ependymoma cases (not shown). Taken together, these data indicated that NHERF1 organizes complexes mainly with moesin and PTEN at the apical PM of polarized structures from ependymal neoplastic cells.

### NHERF1 is a diagnostic marker for ependymoma

To determine whether NHERF1 can be used as a diagnostic marker of ependymal tumors, a multi-institutional effort assembled a total of 113 primary brain tumors consisting of ependymomas, anaplastic ependymomas, and lower grade ependymal tumors, as well as miscellaneous other tumors considered in the differential diagnosis (Table 1). Although we focused our attention on the diagnosis of adult cases, smaller subsets of pediatric cases were also included for comparison. Patient demographics, as well as the localization of tumors, are presented in Table 1.

**Table 1:** **NHERF1 in ependymal tumors and in other tumors considered in the differential diagnosis**

**Diagnosis**	**Patients**		**Site**	**NHERF1 microlumen positivity**			
	**No. cases gender**	**Mean age (range)**		**Diffuse**	**Focal**	**Total/Site**	**Total**
Ependymoma	34^1^	44.6 (12^1^-74)	ST: 5^1^	5 (100%)		5 (100%)	34 (100%)
	20M, 14F		PF: 8	8 (100%)		8 (100%)	
			SC:21	18 (85%)	3^2^ (15%)	21 (100%)	
Adult Anaplastic ependymoma	9	33 (23–49)	ST: 7	2 (28%)	2 (28%)	4 (57%)	6 (67%)
	5M, 4F		PF: 1	1 (100%)	0	1 (100%)	
			SC: 1	0	1 (100%)	1 (100%)	
Pediatric Anaplastic ependymoma	5 4F, 1M	11.8 (6–17)	ST: 3	2 (67%)	1 (33%)	3 (100%)	5 (100%)
			PF: 2	2 (100%)	0	2 (100%)	
Subependymoma	6 4M, 2F	60.5 (40–68)	ST: 5 PF: 1	5 (100%) 1 (100%)	0 0	5 (100%) 1 (100%)	6 (100%)
Mixopapillary ependymoma	5 3F, 2M	45.6 (34–65)	SC: 5	0	3 (60%)	3 (60%)	3^3^ (60%)
Glioblastoma	15^1^ 7M, 8F	54.2 (12^1^-76)	ST: 13 PF: 2^1^	0 0	3 (23%) 0	3 (23%) 0	3 (20%)
AT/RT	2 2F	0.75 (0.4-1.1)	PF: 2	0	0	0	0%
Medulloblastoma	6 5M, 1F	24 (8–63)	PF: 6	0	0	0	0%
Diffuse gliomas^4^	22 16M, 6F	46.5 (27–78)	ST: 22	0	0	0	0%
Pilocytic astroc.	4 3M, 1F	31.5 (18,61)	ST:1 PF: 2 SC: 1	0	0	0	0%
Schwannoma	5 3M, 2F	46 (29–56)	PF: 4 SC: 1	0	0	0	0%

M, male; F, female; ST, supratentorial; PF, posterior fossa; SC, spinal cord.

^1^1pediatric case.

^2^2 tanycytic and 1 giant cell ependymoma.

^3^All myxopapillary ependymomas had focal NHERF1 membranous staining and positive canals.

^4^Diffuse gliomas comprise oligodendroglioma WHO grade II (n = 5) and III (n = 1), oligoastrocytoma WHO grade II (n = 8) and III (n = 4), and astrocytoma WHO grade II (n = 1) and III (n = 3).

**Figure 4:**
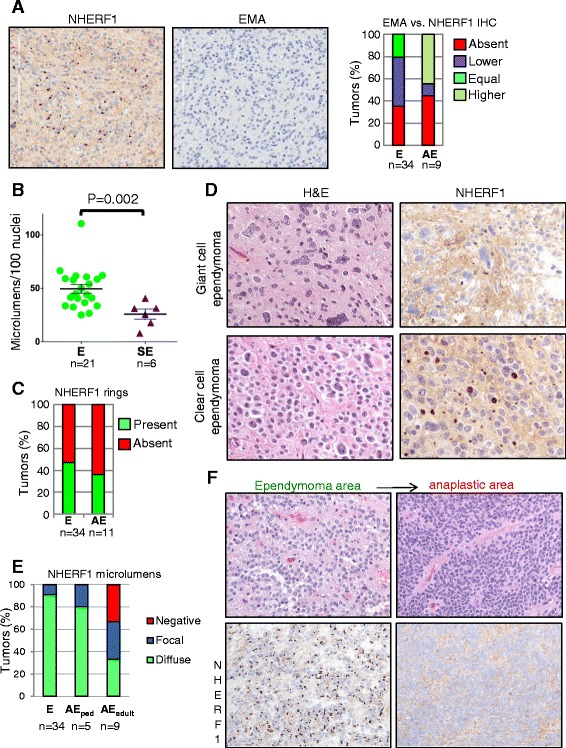
**NHERF1 is a marker for ependymoma. A**. Comparative IHC with NHERF1 and EMA antibodies on serial sections from an ependymoma case shows microlumen detection only by NHERF1. The EMA versus NHERF1 staining was similarly performed in all the ependymomas (E) and in the NHERF1-positive anaplastic ependymomas (AE), and the quantification is shown in the graph. **B**. Quantification of microlumen density in ependymal tumors. E, ependymoma; SE, subependymoma. **C**. Quantification of the ring-like structures labeled by NHERF in ependymoma (E) and anaplastic ependymoma (AE) tumors. **D**. IHC with NHERF1 antibody in ependymoma variants shows microlumen labeling. **E**. Extent of NHERF1 microlumen labeling in ependymoma (E), pediatric anaplastic ependymoma (AE_ped_) and adult anaplastic ependymoma (AE_adult_) illustrates significant NHERF1 loss in the latter. **F**. NHERF1 IHC of an anaplastic ependymoma case containing areas of classical ependymoma morphology (left) and areas of anaplasia (right) shows almost complete loss of NHERF1-labeled microlumens from the anaplastic component.

All 34 ependymoma cases in our series showed NHERF1 expression in microlumens, either in a diffuse pattern (31 of 34 cases), or more rarely, in a focal distribution (3 of 34 cases). The diffuse NHERF1 microlumen pattern was also present in 35.3% and 44.1% of cases with negative or lower epithelial membrane antigen (EMA) staining, respectively (Figure 4A and Additional file 1: Figure S5), indicating a higher sensitivity of NHERF1 for microlumen detection in ependymoma. The density of microlumens was quantified in tumors with diffuse NHERF1 expression and showed approximately 1 microlumen/2 nuclei (Figure 4B and Additional file 1: Figure S6) in the majority of the tumors. A sparse diffuse NHERF1 expression was also observed in some ependymoma cases (as in Figure 4A), with microlumen density similar to that observed in subependymoma cases. We also quantified the presence of ring-like structures and found 47% and 36.3% of ependymomas and anaplastic ependymomas, respectively, to contain these specific structures (Figure 4C). Notably, these structures were detected in only 31% of ependymomas by EMA IHC in a previous study [21]. Focal NHERF1 labeling was observed in two cases of the tanycytic ependymoma subtype, although several other tanycytic ependymomas showed diffuse NHERF1 pattern, and in one case of the giant cell ependymoma subtype (Figure 4D). Clear cell ependymomas showed diffuse NHERF1 microlumen labeling (Figure 4D).

WHO grade I subependymomas showed sparse diffuse NHERF1 microlumen labeling in 100% of the cases, with a density of 1 microlumen/4 nuclei (Figure 4B and Additional file 1: Figure S7). Although microlumen density was significantly lower than in ependymoma, the diffuse presence of microlumens is consistent with an ependymal origin of subependymomas.

Myxopapillary ependymomas are WHO grade I ependymal tumors with good prognosis arising with highest frequency in the cauda equina. NHERF1 labeling showed a distinctive pattern consisting mainly of canals, membranous staining, and focal areas of microlumens (Additional file 1: Figure S8 and Table 1).

Our analysis of WHO grade III anaplastic ependymomas showed a greater degree of NHERF1 expression in pediatric cases in comparison to adult tumors. NHERF1 microlumens were seen in 100% of pediatric cases, primarily with a diffuse pattern, while adult cases showed an overall 67% NHERF1 microlumen positivity with reactivity equally divided between diffuse and focal (Table 1, Figure 4E). In anaplastic tumors with foci of classical ependymoma morphology, an abrupt NHERF1 expression loss was noted in the anaplastic component of the tumor (Figure 4F and Additional file 1: Figure S9), suggesting loss of differentiation in these advanced tumors. Interestingly, EMA perinuclear reactivity was maintained in the anaplastic areas (Figure 4A graph and Additional file 1: Figure S9), reminiscent of the upregulation of EMA, a transmembrane glycoprotein also known as Mucin 1, in a series of epithelial cancers. Even if this reactivity corresponds most likely to Golgi rather than to microlumen staining, it is sometimes difficult to distinguish between these patterns. The four cases in which EMA was retained in anaplastic areas but NHERF1 was negative were scored as higher EMA (Figure 4A graph and Additional file 1: Figure S9). EMA staining was absent in four of the NHERF1-positive cases, two of which had only focal NHERF1 microlumen labeling (Figure 4A graph).

The specificity of NHERF1 microlumen pattern as diganostic marker for ependymal tumors was assessed by screening 54 tumors of different origin that are typically considered in the differential diagnosis (Table 1). For posterior fossa pediatric tumors, medulloblastomas consistently lacked NHERF1 polarity structures and the two cases of atypical teratoid/rhabdoid tumors screened were negative as well. For adult posterior fossa and spinal cord tumors, schwannomas were negative for NHERF1 polarity structures. Similarly, NHERF1 polarity structures were absent in glial tumors such as pilocytic astrocytoma, oligodendroglioma, mixed oligoastrocytoma and anaplastic astrocytoma. Most glioblastoma cases were negative for NHERF1 microlumen labeling, however, 20% showed focal microlumen formation (Additional file 1: Figure S10). No ring-like or other polarity structures were labeled by NHERF1 in these cases.

## Discussion

The pathologic diagnosis of ependymoma is based on H&E histologic examination and confirmation of the neoplastic origin by glial fibrillary acidic protein IHC. True ependymal rosettes and canals are obvious histologic features of ependymoma that occur in a minority of cases. Microlumens, the putative precursor of true rosettes, are more prevalent and are detected traditionally by EM. As EM is expensive, time-consuming and restricted to only few centers, EMA IHC is used routinely as an alternative to EM for microlumen detection. Unfortunately, EMA is not a reliable diagnostic tool due to its low detection sensitivity, as in our series, and to its reported decreased specificity for ependymoma [21]. We show here that NHERF1 IHC has a high sensitivity and specificity for microlumen detection in ependymal tumors, and therefore can be used reliably as a diagnostic marker in these tumors. We have also identified moesin in ependymal polarity structures, however, the low affinity of the moesin antibody and its labeling of blood vessels indicate that NHERF1 is a superior diagnostic marker. The lower grade ependymal tumors, including subependymoma and ependymoma, consistently showed NHERF1 microlumen labeling, usually with diffuse pattern. Labeling in anaplastic varied by age: all pediatric cases were NHERF1-positive, generally with diffuse reactivity, while only two-thirds of adult cases were NHERF1-positive, either diffusely or focally. The presence of areas of classical ependymoma morphology with abundant NHERF1 staining in otherwise anaplastic tumors supports the diagnosis in these advanced tumors. To our knowledge, NHERF1 IHC represents the most sensitive method for microlumen detection in ependymoma.

Due to the lack of effective chemotherapy regimens, recent efforts have been directed towards understanding the pathogenesis of ependymoma. Extensive mRNA microarray and CGH analyses showed that ependymomas are heterogenous tumors that, depending on their location –spinal, supratentorial, or in the posterior fossa - show different molecular signatures [30,31]. Interestingly, NHERF1, a marker of apical PM in normal ependyma, consistently highlighted the microlumens of ependymoma regardless of location, attesting to a common origin for these tumors. The presence of identical NHERF1-labeled microlumens in clusters of normal ependymal cells that do not line the ventricular system raises the possibility of tumor initiation from these clusters. It is thus foreseeable that the different molecular signatures result from the proliferative response of similar ependymal precursor cells to location-specific environmental cues. In this respect, it is noteworthy that defects of ciliogenesis characterize both a subset of posterior fossa ependymomas in children [31] and a series of developmental posterior fossa deficits [32,33], pointing to common molecular pathways for both posterior fossa abnormalities.

Apico-basal cell polarity is a morphological characteristic disrupted early in the development of epithelial malignancies [34]. We have previously shown that NHERF1 deficiency in mice induces structural abnormalities of the intestinal apical PM [16] that translate into defective epithelial morphogenesis with loss of apico-basal polarity and epithelial-mesenchymal transition in colorectal cancer cells [9,12]. In this study, the presence of hydrocephalus and of ependymal apical PM defects in *NHERF1*-deficient mice translated into the characterization of NHERF1-containing precursor polarized structures in ependymoma. The sensitive detection of microlumens by NHERF1 antibody revealed loss of these structures in anaplastic foci present in some WHO grade II ependymomas and a drastic reduction in adult anaplastic ependymoma, most likely due to lack of differentiation of the constituent anaplastic cells. Beside its structural role, NHERF1 has been implicated in oncogenic signaling, especially in the phopshoinositide 3-OH kinase (PI3K)-Akt and Wnt-β-catenin pathways [10,11,27,35]. In glioblastoma, NHERF1 loss from the PM has been shown to displace PTEN from the PM and consequently activate PI3K-Akt pathaway [14]. Similarly, the loss of NHERF1 and associated proteins from the PM of ependymal polarity structures in anaplastic ependymoma is prone to result in PTEN cytoplasmic displacement and activation of the PI3K-Akt pathway. Thus, in an analogous manner to other cancers [12,14,36], the regulation of morphogenesis and cell growth by NHERF1 subcellular localization emerges also in ependymal oncogenesis, with direct translation to the diagnosis of ependymal tumors.

## Additional file

Additional file 1: Figure S1. Subclinical hydrocephalus in *NHERF1*-deficient mice. **Figure S2.** NHERF1 expression in the CNS of mice. **Figure S3.** Disruption of the ependymal layer in *NHERF1*-deficient mice. **Figure S4.** Expression of β-catenin and YAP1 in ependymoma. **Figure S5.** Comparative NHERF1 and EMA labeling in ependymoma. **Figure S6.** Quantification of NHERF1 microlumen density in ependymoma. **Figure S7.** NHERF1 expression in subependymoma. **Figure S8.** NHERF1 expression in myxopapillary ependymoma. **Figure S9.** Comparative NHERF1 and EMA labeling in anaplastic ependymoma. **Figure S10.** NHERF1 expression in glioblastoma.
