# Supplementary material for: NHERF1/EBP50 is an organizer of polarity structures and a diagnostic marker in ependymoma
Source: Acta Neuropathol Commun. 2015 Mar 8;3:11. doi: 10.1186/s40478-015-0197-z (PMC4352254; doi:10.1186/s40478-015-0197-z)
Supplement: Additional file 1: Figure S1. — Subclinical hydrocephalus in NHERF1-deficient mice. Figure S2. NHERF1 expression in the CNS of mice. Figure S3. Disruption of the ependymal layer in NHERF1-deficient mice. Figure S4. Expression of β-catenin and YAP1 in ependymoma. Figure S5. Comparative NHERF1 and EMA labeling in ependymoma. Figure S6. Quantification of NHERF1 microlumen density in ependymoma. Figure S7. NHERF1 expression in subependymoma. Figure S8. NHERF1 expression in myxopapillary ependymoma. Figure S9. Comparative NHERF1 and EMA labeling in anaplastic ependymoma. Figure S10. NHERF1 expression in glioblastoma. [file 40478_2015_197_MOESM1_ESM.pdf]

SUPPLEMENTAL FIGURES

A

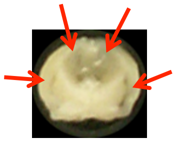

Figure S1. <u>Subclinical hydrocephaly in *NHERF1*-deficient mice.</u> **A.** Dissection of fresh brain from an apparently clinically unaffected 5-week-old *NHERF1*(-/-) male shows bilateral moderate dilatation of lateral ventricles (arrows). **B.** Incidence of subclinical hydrocephaly in 3 litters from *NHERF1*(+/-) parents. Clinically healthy 5-week-old offspring showed mild or moderate hydrocephaly visualized after brain dissection.

B

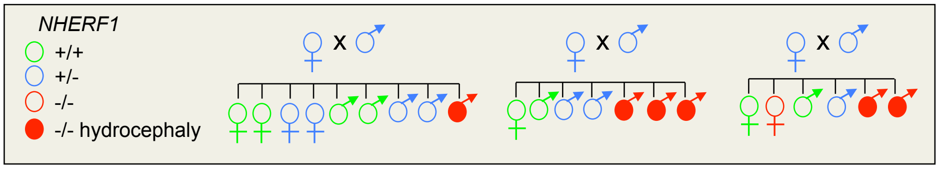

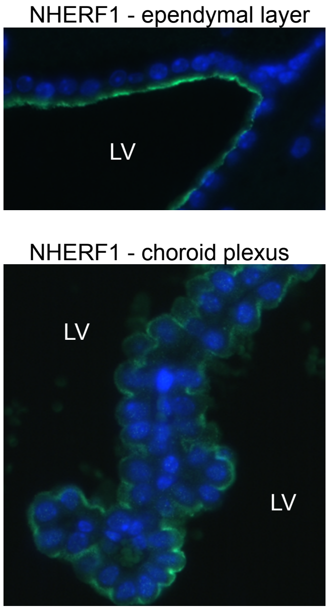

Figure S2. <u>NHERF1 expression in the CNS of mice.</u> Immunofluorescence analysis of formalin-fixed paraffin-embedded brain sections from *NHERF1*(+/+) mice with NHERF1 antibody (green) and DAPI (blue) for nuclei labeling. LV, lateral ventricle. Note apical PM expression of NHERF1 in both ependymal and choroid plexus cells and lower cytoplasmic expression in choroid plexus cells.

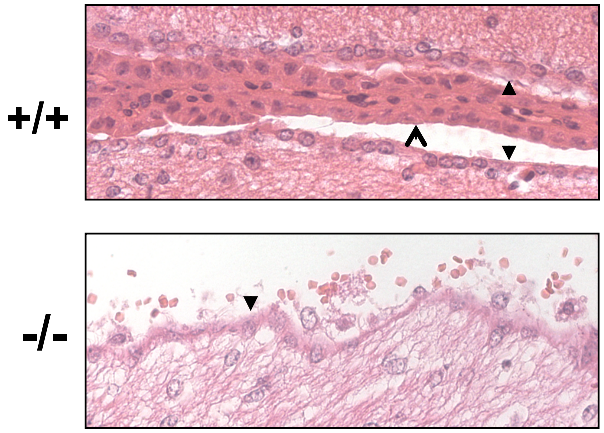

Figure S3. <u>Disruption of the ependymal layer in *NHERF1*-deficient mice.</u> H&E staining of formalin-fixed paraffin-embedded brain sections from the 5-week-old littermates analyzed in Fig. 1A shows thinning of the ependymal layer (closed arrowheads) with cell detachment and rarefaction of the subependymal neuropil in *NHERF1*(-/-) mice with severe hydrocephaly. The choroid plexus is indicated with open arrowhead.

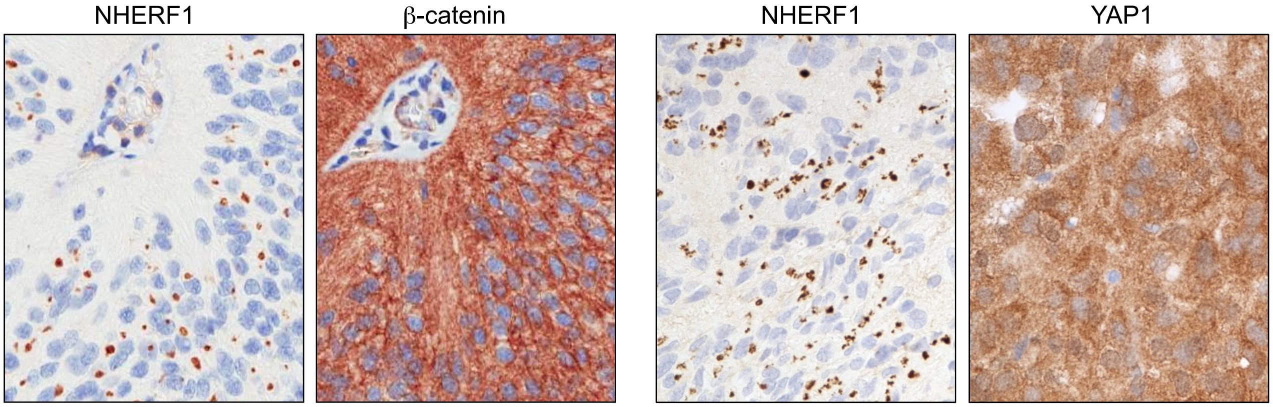

Figure S4. Expression of β-catenin and YAP1 in ependymoma. NHERF1 PDZ2 domain ligands β-catenin and YAP1 show cytoplasmic staining without apparent NHERF1 co-localization in microlumens. YAP1 shows also nuclear staining. The same IHC pattern is present in anaplastic ependymoma.

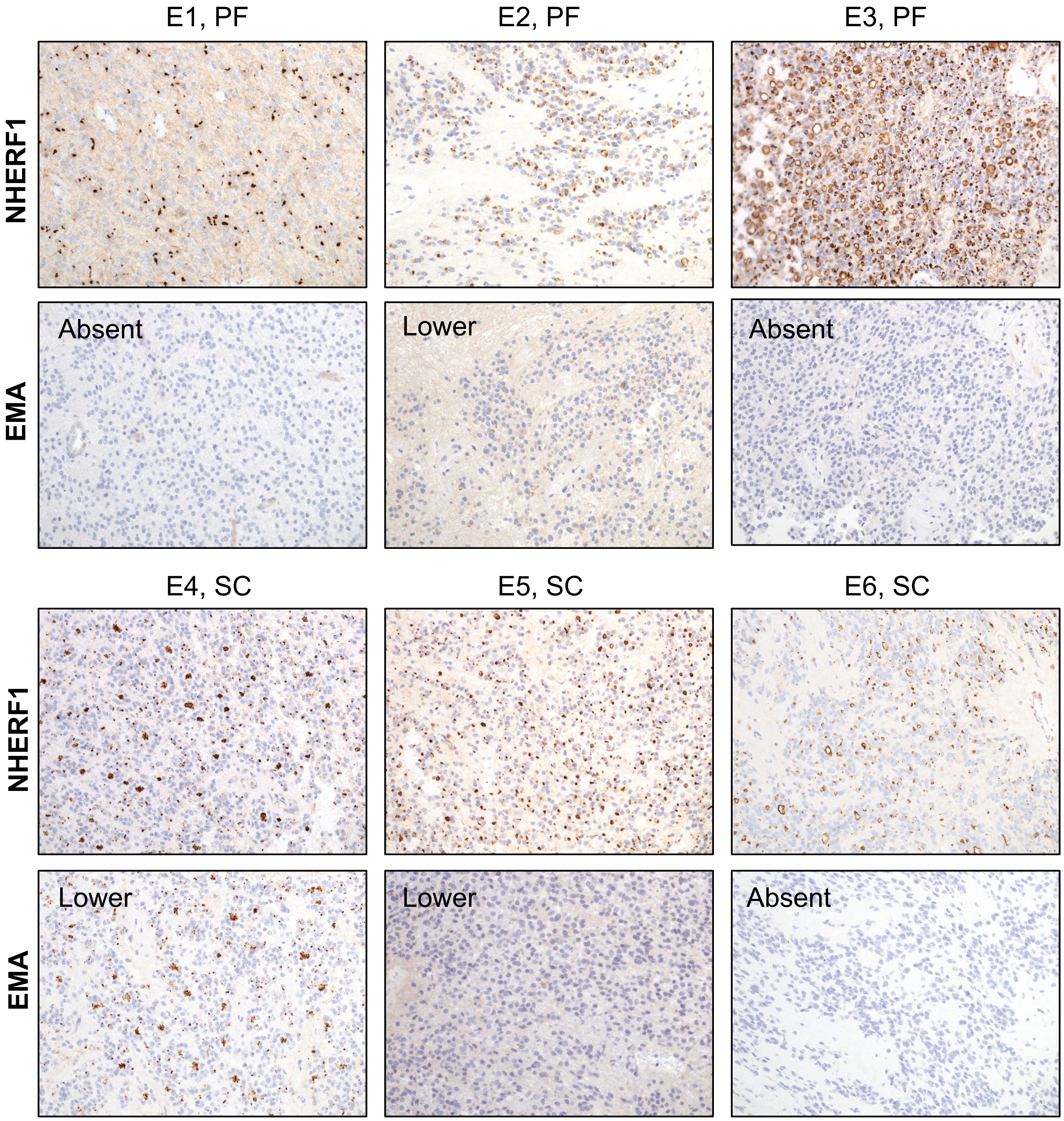

Figure S5. Comparative NHERF1 and EMA labeling in ependymoma. NHERF1 and EMA IHC were performed on serial sections in the cases indicated in Fig. 4A. Six different examples comparing NHERF1 staining with either absent or lower EMA staining are shown for posterior fossa (PF) or spinal cord (SC) ependymomas. Images of the same field were acquired at 20x magnification.

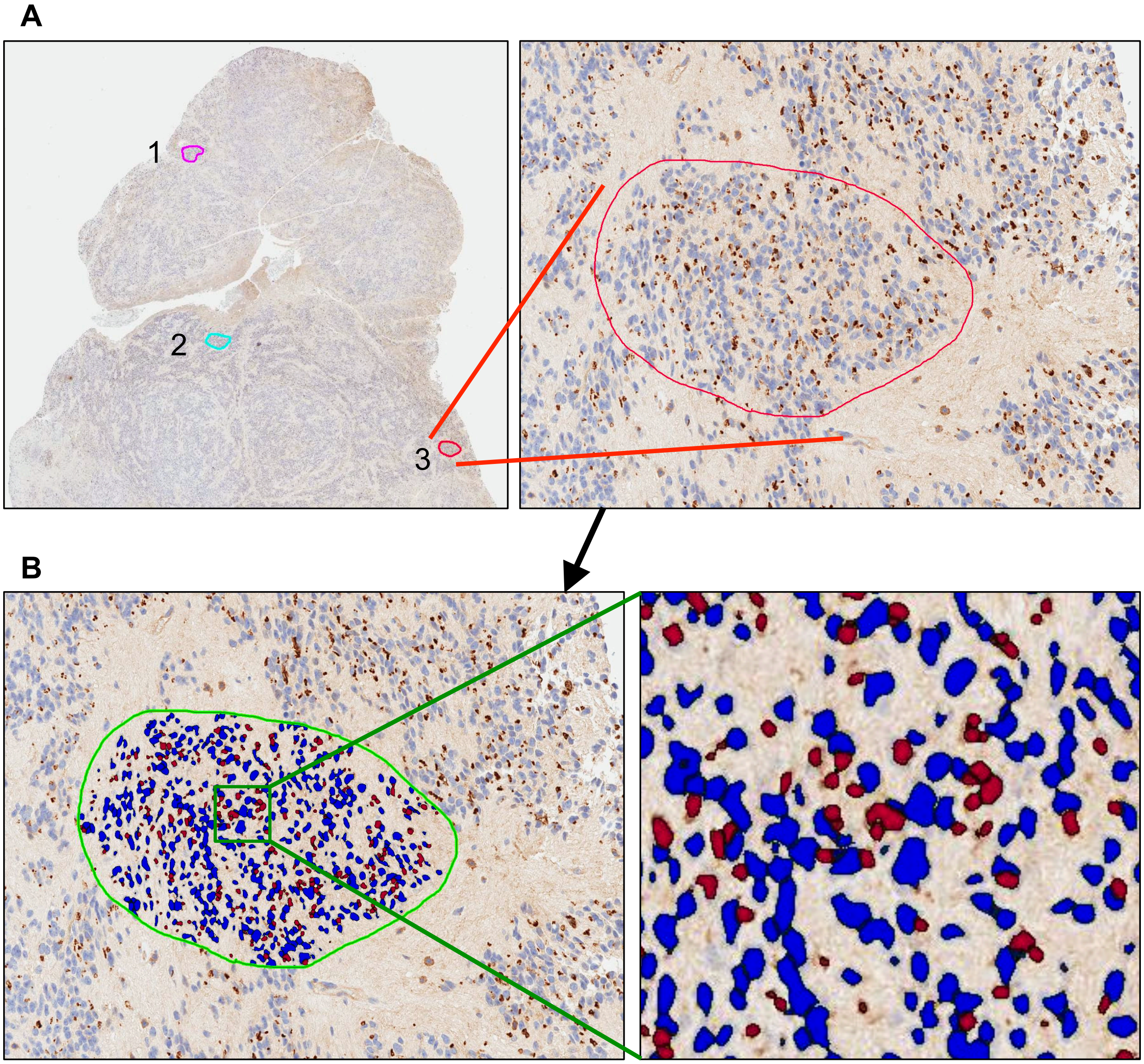

Figure S6. Quantification of NHERF1 microlumen density in ependymoma. Images were acquired at 20x magnification, with Aperio Scanscope CS2 whole slide image system and analyzed by ImageScope software, version 12.1.0.5029. **A.** Three tumor areas were analyzed from each slide at 20x magnification. **B.** The Nuclear algorithm was used for object recognition in order to obtain the primary output represented by the number of NHERF1 positive microlumens (brown dots) and number of negative nuclei (blue dots).

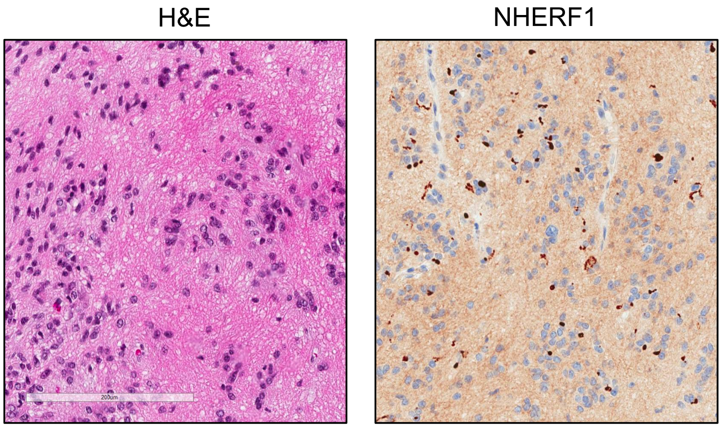

Figure S7. NHERF1 expression in subependymoma. Subependymomas show sparse and diffuse dot-like NHERF1 staining of microlumes.

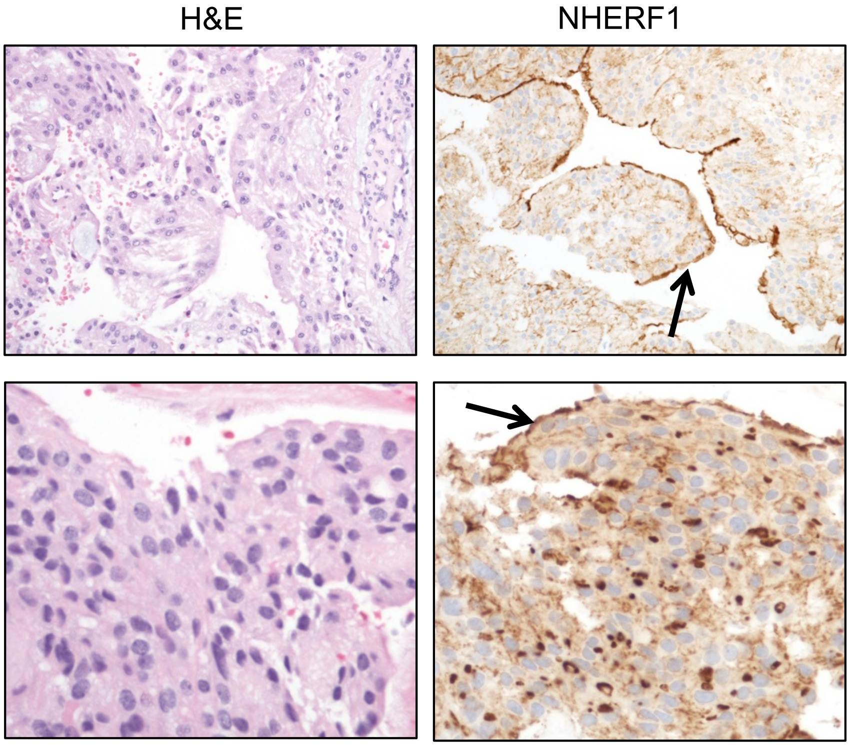

Figure S8. NHERF1 expression in myxopapillary ependymoma. H&E staining and NHERF1 IHC in two cases of myxopapillary ependymoma showing the characteristic staining of canals (arrows) and the less frequent dot-like perinuclear staining of microlumens in some areas in the second case (lower panels).

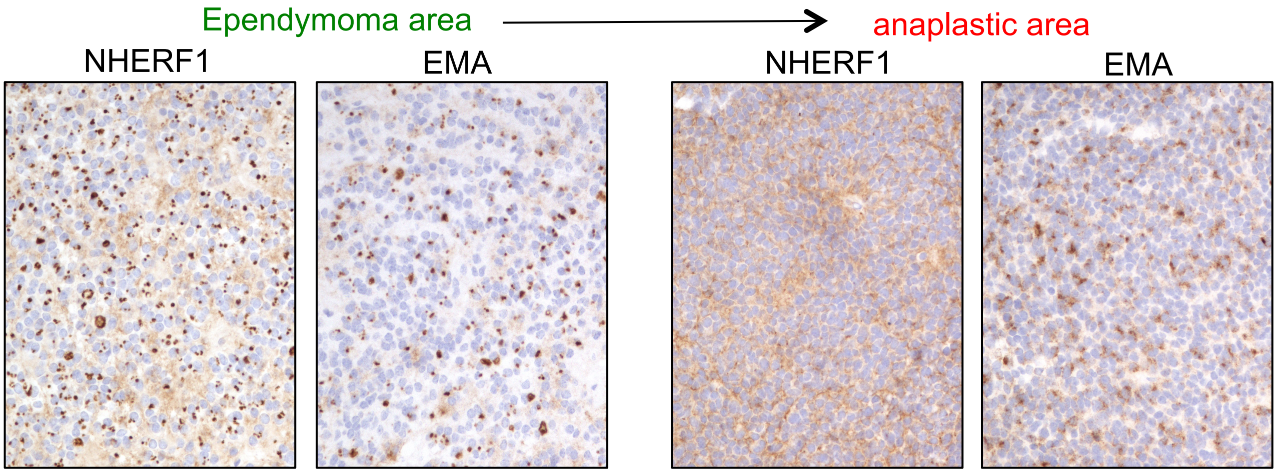

Figure S9. Comparative NHERF1 and EMA labeling in anaplastic ependymoma. IHC with NHERF1 and EMA antibodies of serial sections from the anaplastic ependymoma case in Fig. 4F shows lower EMA staining in the differentiated ependymoma-like areas and higher EMA staining in the anaplatic areas. Images of the same field were acquired at 40x magnification and the field with highest EMA staining is shown in both areas. Note a more diffuse quality of the perinuclear EMA staining in the anaplastic areas, compatible with Golgi staining. Note also increased NHERF1 cytoplasmic staining and lack of dot-like staining in the anaplastic areas compared to differentiated ependymoma-like areas.

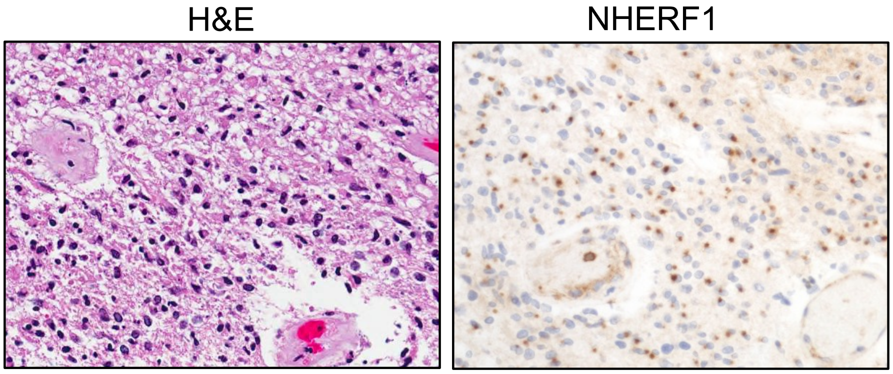

Figure S10. NHERF1 expression in glioblastoma. NHERF1 IHC in glioblastoma usually lacks dot-like microlumen positivity. However, focal NHERF1 microlumen labeling can be present in rare cases, as shown here.
